# Electronic patient-reported outcomes and machine learning in predicting immune-related adverse events of immune checkpoint inhibitor therapies

**DOI:** 10.1186/s12911-021-01564-0

**Published:** 2021-06-30

**Authors:** Sanna Iivanainen, Jussi Ekstrom, Henri Virtanen, Vesa V. Kataja, Jussi P. Koivunen

**Affiliations:** 1 Department of Oncology and Radiotherapy, Oulu University Hospital and MRC Oulu, OYS, P.B. 22, 90029 Oulu, Finland; 2 Kaiku Health Oy, Helsinki, Finland

**Keywords:** ePRO, Machine learning, Prediction model, irAE, Immune checkpoint inhibitor, Prognosis

## Abstract

**Background:**

Immune-checkpoint inhibitors (ICIs) have introduced novel immune-related adverse events (irAEs), arising from various organ systems without strong timely dependency on therapy dosing. Early detection of irAEs could result in improved toxicity profile and quality of life. Symptom data collected by electronic (e) patient-reported outcomes (PRO) could be used as an input for machine learning (ML) based prediction models for the early detection of irAEs.

**Methods:**

The utilized dataset consisted of two data sources. The first dataset consisted of 820 completed symptom questionnaires from 34 ICI treated advanced cancer patients, including 18 monitored symptoms collected using the Kaiku Health digital platform. The second dataset included prospectively collected irAE data, Common Terminology Criteria for Adverse Events (CTCAE) class, and the severity of 26 irAEs. The ML models were built using extreme gradient boosting algorithms. The first model was trained to detect the presence and the second the onset of irAEs.

**Results:**

The model trained to predict the presence of irAEs had an excellent performance based on four metrics: accuracy score 0.97, Area Under the Curve (AUC) value 0.99, F1-score 0.94 and Matthew’s correlation coefficient (MCC) 0.92. The prediction of the irAE onset was more difficult with accuracy score 0.96, AUC value 0.93, F1-score 0.66 and MCC 0.64 but the model performance was still at a good level.

**Conclusion:**

The current study suggests that ML based prediction models, using ePRO data as an input, can predict the presence and onset of irAEs with a high accuracy, indicating that ePRO follow-up with ML algorithms could facilitate the detection of irAEs in ICI-treated cancer patients. The results should be validated with a larger dataset.

*Trial registration* Clinical Trials Register (NCT3928938), registration date the 26th of April, 2019

## Background

In recent years, there has been a remarkable development in cancer immune checkpoint inhibitor (ICI) therapies. ICIs have become the first line treatments in several malignancies [1–12]. However, ICIs are associated with unique immune related adverse events (irAEs). These toxicities can arise from various organ systems, and, at any time point without temporal connection to the therapy which makes these more unpredictable than AEs with traditional cancer therapies. irAEs may also persist or appear in a similar manner after ICI discontinuation while immune-mediated toxicity seems to be independent of the dose and duration of the given anti-PD-(L)1 treatment [13–15]. Even though irAEs can be severe and even life threatening, if caught and treated early, most of them are reversible [16]. Thus, early detection of irAEs could result in an improved safety of the treatment and better quality of life (QoL).

Artificial intelligence (AI) based analytics have gained growing interest in the field of cancer care. Deep learning systems have shown promising results especially in cancer diagnostics [17]. AI based methods can be used to analyze vast data pools to create predictive and prognostic analytics for generating value-based healthcare assets. In addition, recent data shows that ML algorithms could identify patients with cancer who are at risk of short-term mortality [18]. The possibility of creating truly individual prognostic assessments could facilitate more timely conversations between patients and health care personnel (HCP) regarding goals and values.

Due to the unique nature of irAEs, there is a need for all-comprising assessment, grading, and long-term surveillance of patients’ symptoms. Patient reported outcomes (PROs) consist of health-related questionnaires complete by the patients, which can capture symptoms and signs. Evolving data suggests that electronic (e) PRO tools could be one solution for optimizing patient surveillance during and after ICI therapies [19, 20]. ePROs combined with other clinical data could be used to develop machine learning (ML) based prediction models to better foresee irAEs. irAEs can be demanding to distinguish from acute infections or other cancer related symptoms and early detection may be sacrificed with seldom treatment visits at the care unit (up to 6 weeks). ML models could provide a tool to enhance both the irAE diagnosis and early detection and facilitate medically relevant interaction between the patient and HCP.

We have previously shown that the real-world symptom data collected with Kaiku Health ePRO tool on cancer patients receiving ICI therapy aligns with the data from clinical trials, and that correlations between different symptoms occur, which might reflect therapeutic efficiency, side effects, or tumor progression [20, 21]. We first explored the possibilities of ML based prediction models on ePROs to create prediction models of symptom continuity of cancer patients receiving ICIs, and showed that it is feasible [22]. Based on our previous work on ML modelling, and the ePRO symptom correlations, we speculated that if symptoms can predict irAEs, symptoms could work as a surrogate to irAEs. Thus, we hypothesized that a ML based prediction model for irAEs of cancer patients receiving ICIs could be created based on ePRO symptom data coupled with clinical data.

In this study, anonymized and aggregated ePRO data collected with the Kaiku Health ePRO tool, in addition to prospectively collected irAE data containing the initiation and end dates, CTCAE class, nature and severity of irAEs, was used to train and tune prediction models built using an open source Python library XGBoost (extreme gradient boosting algorithm) for the detection of the presence and onset of irAEs [23–25].

## Methods

### Patients

The study subjects (n = 34) consisted of patients recruited to the prospective KISS trial investigating ePRO follow-up on cancer patients receiving ICIs [19]. In brief, the trial included patients with advanced cancers (non-small cell lung cancer, melanoma, genito-urinary cancers and head and neck cancers) treated with anti-PD-(L)1s in outpatient settings with the availability of internet access and email. At the initiation of the treatment phase (within 0–2 weeks from the 1st anti-PD-(L)1 infusion), patients received an email notification to complete the baseline electronic symptom questionnaire of 18 symptoms and weekly thereafter until treatment discontinuation or 6 months of follow-up. Data on the irAEs (nature of AE, date of onset and resolving, dates of change in AE severity, and the highest grade based on CTCAE classification) were prospectively collected in the trial.

The study was approved by Pohjois-Pohjanmaan sairaanhoitopiiri (PPSHP) ethics committee (number 9/2017), Valvira (number 361), and details of the study are publicly available at clinicaltrials.gov (NCT3928938). The study was conducted in accordance with the Declaration of Helsinki and Good Clinical Practice guidelines.

### Prediction models

The aim of this study was to create a model for predicting the presence (is the predicted irAE truly an irAE) and onset (is an irAE developing) of irAEs based on evolving patient-reported symptoms collected digitally in prospective manner from cancer patients receiving ICI therapies. For both modelling cases, the output of the prediction model is a continuous value [0—1] depicting the probability for the positive event, i.e., presence or onset of irAEs. With a classification threshold (0.5 was used with both models), the continuous probabilities were converted into binary outcomes, i.e., when the predicted probability for the positive event is larger than 0.5, prediction is labeled positive (irAE onsetting or present), and if less than 0.5, then negative (irAE not onsetting or present). Hence, the modelling methodology used in this study follows a general framework of binary classification in machine learning (ML). The first dataset included 820 filled symptom questionnaires from from 34 ICI treated cancer patients in outpatient settings, comprising 18 monitored symptoms collected using the Kaiku Health digital platform. The second dataset included physician-confirmed prospectively collected irAE data in the eCRFs of the KISS trial from those 34 patients, containing the initiation and end dates, CTCAE class, nature (colitis, diarrhea, arthritis, rash, hyperglycemia, neutropenia, pneumonitis, itching, cholangitis, mucositis, hypothyreosis, hepatitis) and severity of 26 irAEs. The timelines of ePROs and irAEs were synchronized according to dates. Of note, some patients might have experienced multiple irAEs, thus, the incidence of irAEs in this patient cohort was ~ 40% (n = 14). Multiple observations across same patients were used to create a timeline of irAEs, however, in every time point analyzed, the parameters differ comprising a new sample. Furthermore, the gradient boosting trees-algorithm used can handle intercorrelated observations or features.

Two ML models were built using an open source Python library XGBoost, which offers a widely used high-performance implementation of gradient boosting, an established algorithm suitable for classification problems. Gradient boosting is an ensemble-learning algorithm, i.e., it is an ensemble of many, usually tens or hundreds of decision trees. These decision trees, i.e., classification trees, are weak learners, but when combined using gradient boosting approach, they form a strong learner capable of capturing complex relationships in the training data. By combining the ePRO data and the clinical data, the first model was trained to detect the presence and the second model the onset (0–21 days prior to the diagnosis) of irAEs. We also tested several other commonly used ML models, such as logistic regression, elastic-net regression, support vector machines, LightGBM and random forests, but XGBoost had the best performance with the test dataset, and, thus, it was chosen as the model for the study.

The dataset was split into training (70% of the data) and test sets (30% of the data) by random allocation at patient-observation level. The test set was left out from the model training and tuning and was used only to evaluate the model performance. The hyperparameter tuning for both prediction models was done using grid search with repeated, stratified fivefold cross-validation with five repeats. The model features included patient information including age, sex, and time from the treatment initiation, and ePRO data from 18 monitored symptoms. From the symptom data, three past values, linearly scaled based on the time difference from the latest report, and the latest change in symptom severity were included as features for each symptom. This yielded 75 features in total for both models.

The prediction performance of the models was evaluated using four commonly used metrics: accuracy, Area Under the Curve (AUC), F1-score and Mathew’s correlation coefficient (MCC), which are described shortly next. Accuracy describes how many predictions were correct as percentages, and 100% indicates a perfect classification. AUC is a performance metric for binary classification ranging from 0 to 1. F1-score is the weighted average of precision (how many of the cases predicted as positive are actually positive) and recall (how many of the positive cases are detected) which gets values between 0 and 1. Matthew’s correlation coefficient (MCC) summarizes all possible cases for binary predictions: true and false positives, and true and false negatives. MCC is also suitable for analyzing imbalanced datasets, where other class is much rarer than the other. MCC can be considered as a correlation coefficient between the observed and the predicted classifications and it gets values between − 1 and 1, where 1 is a perfect classification, 0 is random guessing and − 1 indicates a completely contradictory classification.

## Results

 In this patient group, the longest irAE lasted for 799 days, and the shortest two days while median duration was 61 days. The model trained to predict the presence of irAEs had an excellent performance with the test dataset. According to the accuracy score, both models performed at a very good level. The accuracy score for the prediction of the presence of irAEs was 0.97, and 0.96 for the onset of irAEs. The AUC values (0.99 for the presence of irAEs and 0.93 for the onset of irAEs) suggested a good quality level of the model performance. The F1-score (0.94) indicates that the model was accurate in the prediction of the presence of irAEs. However, F1-score for the prediction of the onset of irAEs was somewhat lower, 0.66. According to the MCC values, the model performed well in predicting the presence of irAEs (0.92) while the accuracy in predicting the onset of irAEs was lower, 0.63. The performance metrics for the ML models are presented in Table 1.

**Table 1:** Performance metrics for the prediction models for the presence and onset of irAES

	Presence of irAEs	Onset of irAEs
Accuracy	0.97	0.96
AUC	0.99	0.93
F1-score	0.94	0.67
MCC	0.92	0.64

**Fig. 1:**
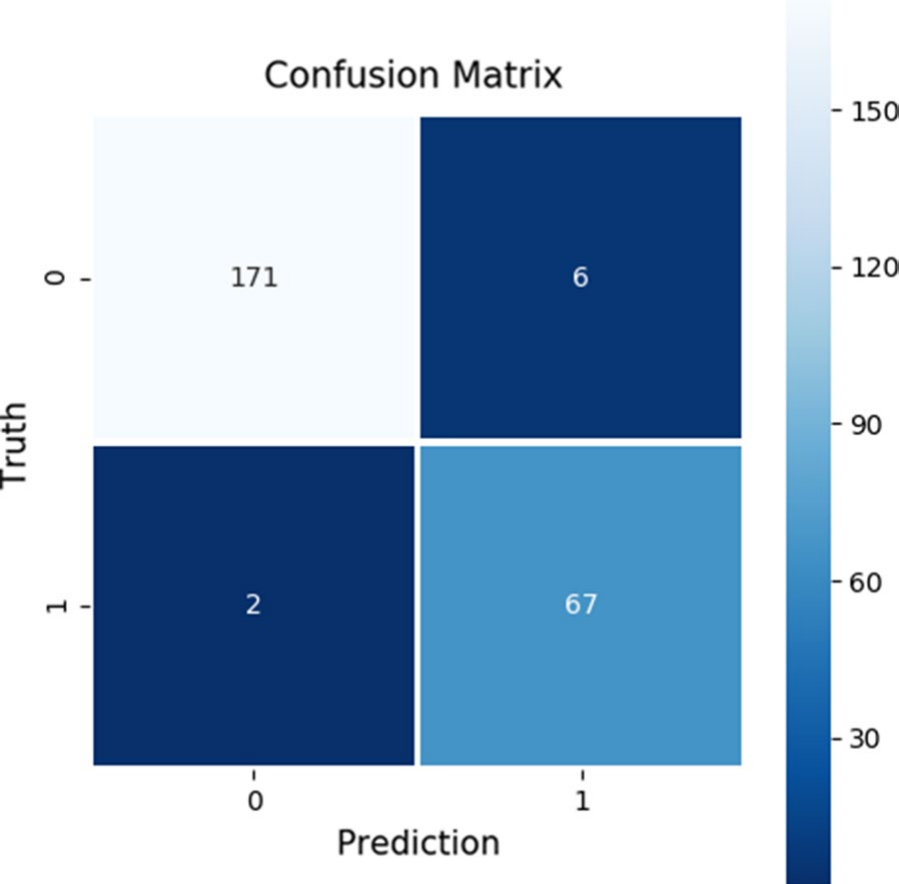
The confusion matrix for predicting the presence of irAEs

**Fig. 2:**
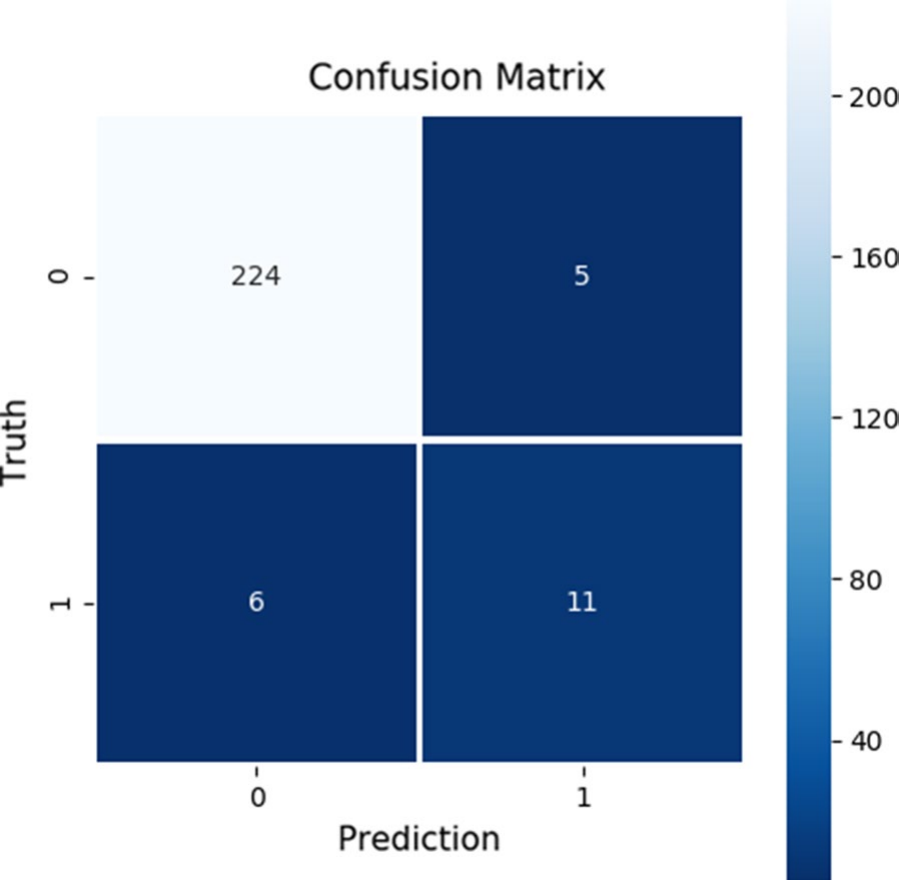
The confusion matrix for predicting the onset of irAEs

Figure 1 presents the confusion matrix for predicting the presence of irAEs and Fig. 2 for predicting the onset of irAEs. As is evident from Fig. 1, the prediction performance for the presence of irAEs was excellent as there were only two false negative (the lower left corner) and six false positive (the upper right corner) predictions with the test dataset. The false negatives were identified as the cases where the prediction model did not predict a presence of irAE for a test dataset sample, which was actually positive, i.e., an irAE was present. The false positives, on the other hand, were the cases where the model predicted the presence of irAE for the sample, but the sample was actually negative, i.e., there was no irAE present.

The prediction performance for the onset of irAEs was much lower as can be seen in Fig. 2. There were only 17 positive samples in the test dataset of which 11 were classified correctly as positive and six were classified as negative, thus, being false negatives. In addition, there were five false positives in the test dataset.

**Fig. 3:**
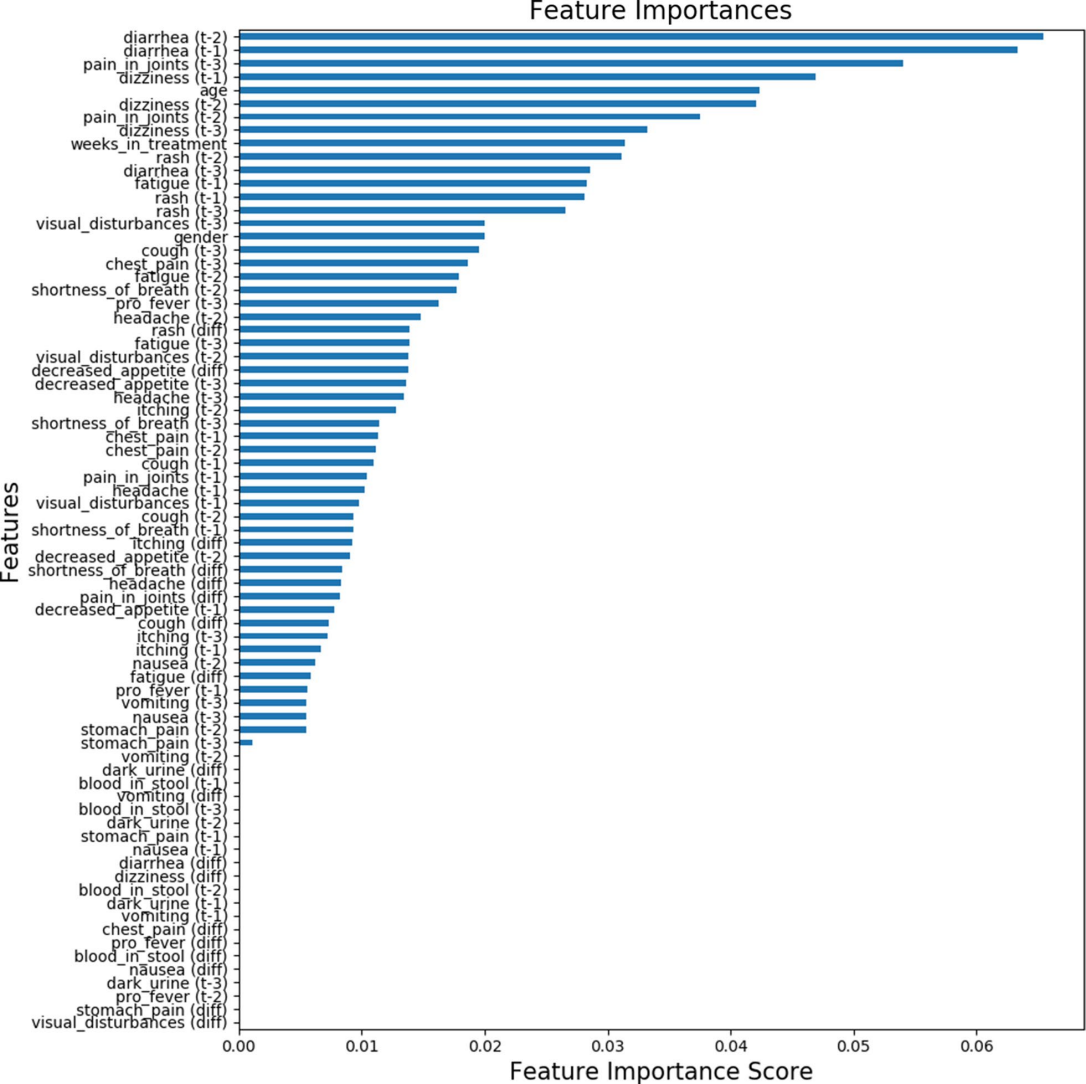
Feature importance plot for the model predicting the presence of irAE

**Fig. 4:**
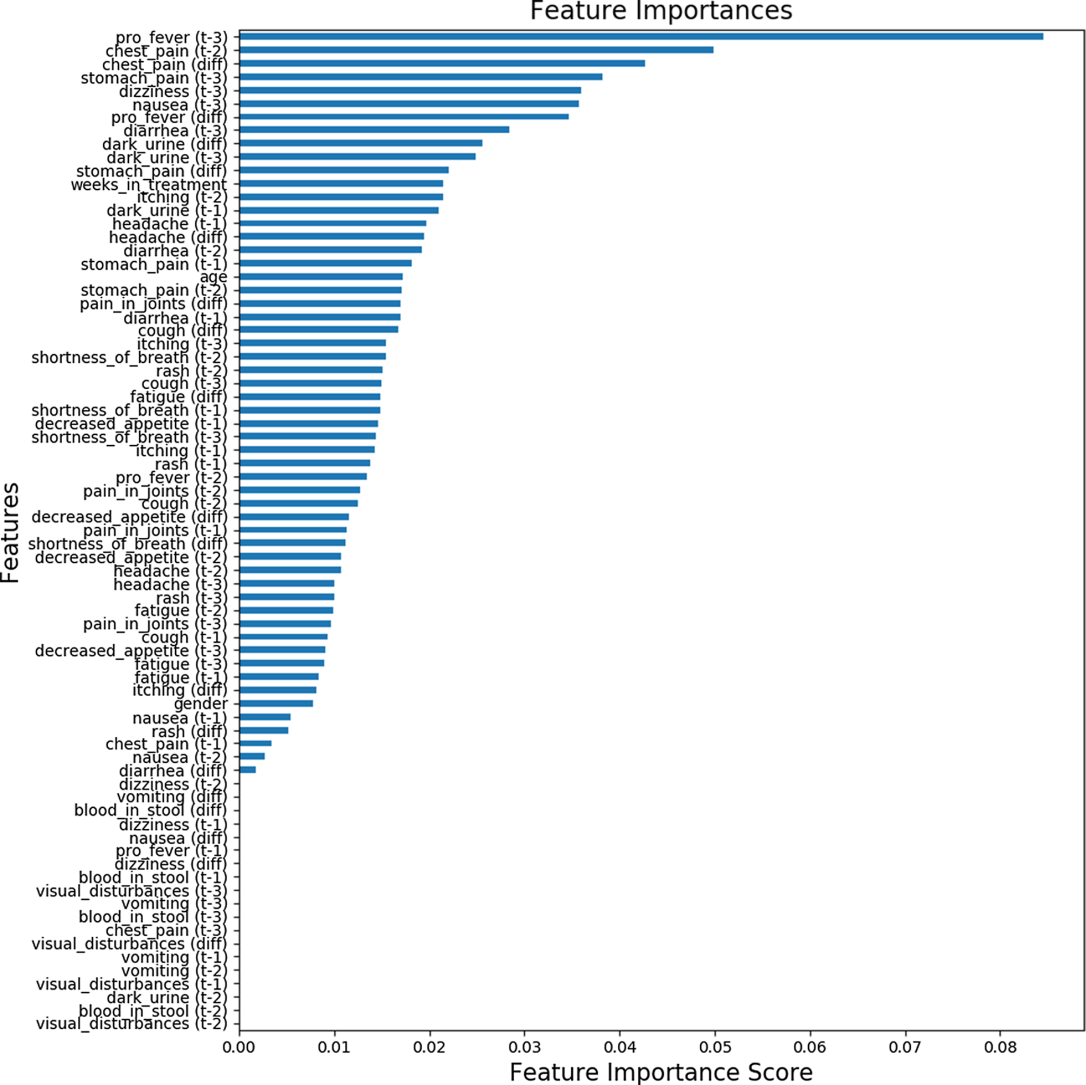
Feature importance plot for the model predicting the onset of irAEs

Figure 3 presents the feature importance plot for the model predicting the presence of irAEs and Fig. 4 for the model predicting the onset of irAEs. The presented feature importances display the relative average improvement in prediction accuracy across all trees in the model where the feature in question is used. The feature importances are relative, i.e., they display how much features contribute to the final prediction relative to each other. A higher value indicates that the feature is more important for generating the prediction compared to a feature with a lower value.

As is presented in Fig. 3, the most important features for predicting the presence of irAEs were related to diarrhea, pain in joints, dizziness, age and time from the treatment initiation (weeks in treatment). According to Fig. 4, for predicting the onset of irAEs, the most important features were related to fever, chest pain, stomach pain, dizziness, nausea, and diarrhea. Furthermore, Fig. 3 reveals that, roughly, two thirds of the features contributed to the predictions and one third was not used. The features, which do not contribute to the predictions, could be removed using feature selection, but it is not mandatory, or does not impact the model performance due to the tree structure of the used algorithm. A similar number of features were utilized in the prediction of the onset of irAEs neither, as is presented in Fig. 4.

## Discussion

In this study, ePRO and irAE data related to advanced cancer patients treated with ICIs were investigated to better understand their potential correlation. The aim was to use prospectively collected patient-derived data and clinical data from treating physicians during ICI treatments to build ML based tools to improve the early detection of irAEs, and ultimately, enhance patient care, QoL and cost-effectiveness of ICIs. The results show that ML models based on the ePRO and structured electronic health care record (EHR) data could accurately predict the presence of irAEs. The ML models had a good level of discrimination in predicting the onset and continuation of irAEs.

The feature importance analysis revealed that diarrhea and pain in the joints, known irAEs with predictive nature, contributed the most to the prediction accuracy when predicting the presence of irAE. Combined with clinical domain knowledge, the importance of these features could be interpreted that if ICI treated cancer patients report these symptoms, especially with a higher severity; they very often reflect the presence of irAE. In contrast, in predicting the future onset of irAE, fever, chest, and stomach pain were the predominant features in the model. All these symptoms are commonly present with cancer patients and demand further assessment and intervention aiming to improve symptom control and QoL. Furthermore, in rare cases, such symptoms could imply an onset of a fatal irAE such as colon perforation, pneumonitis, or myocarditis that require ambulatory interventions. In addition, these symptoms often indicate progressive disease in lung cancer patients or patients with lung metastases, which underlines the importance of prompt diagnostic measures to exclude pseudo-progression with concomitant irAE demanding rapid initiation of an immunosuppressive medication [14].

There are several limitations to this analysis. The most relevant limitation is the low number of study subjects. However, the total amount of reported symptoms is close to 20,000 which increases the value of the data collected. In addition, the data pool was not sufficient large enough to create prediction models for individual irAEs. On the other hand, also in clinical practice the differential diagnostics of irAEs has proven to be demanding due to the generic onset of irAEs. For that, we feel that an early identification of any irAE probably enhances the cancer care especially taking into account the non-specific symptom and irAE correlation, and over time cumulation of multiple irAE on individual patients.

Furthermore, our cohort consisted of ICI-monotherapy treated patients and may not be generalized to patients treated with combination therapies. According to registry data, however, the toxicity profile of ICI-ICI or ICI-chemotherapy compared to ICI-monotherapy differs mainly in the overall incidence rather than in the variety of symptoms [14, 26–30]. In addition, our study provides a proof-of-concept for building ML based prediction models on ePRO data and clinical data and can be further exploited also to create models for the combination therapies of ICIs. The F1-score and the MCC value for the prediction of the onset of irAE were somewhat low. The lower level of performance of the model in predicting the onset of irAEs probably reflects the rather low incidence of irAEs in ICI-monotherapy treated cancer patients. Nevertheless, used modelling methods and approaches were chosen to overcome the issues related to imbalanced data sets, and intercorrelated parameters to minimize such bias. These methods and approaches included, e.g., utilization of sample weights (giving more emphasis on the rare positive samples in model training), utilization of F1 score and MCC as performance metrics and using a regularized tree-based model, XGBoost.

Evolving data show that EHR based predictive algorithms may improve clinicians’ prognostication and decision-making [31, 32]. Oncology-specific ML algorithms based on EHR data have been shown to accurately predict short-term mortality among patients starting chemotherapy [33, 34]. However, utilization of electronic patient-derived symptom data, ePROs, related to ICI therapy toxicity in creating ML algorithms is a novel approach. Furthermore, ePROs have many advantages compared to paper questionnaires such as reducing timely and locational limitations and offering continuous collection of symptoms in a cost-effective manner [35–37]. Thus, based on the results of the study, it is feasible to use ePROs in the development of ML based approaches, such as symptom prediction models to enable the earlier detection of toxicities. Furthermore, we argue that ePRO follow-up combined to ML models for ICI toxicity prediction would optimize the clinical impact of the therapy. Future research will focus on combining other clinical data such as laboratory values to the model to create a criterion-standard prognostic assessment tool to predict ICI related toxicity. Whether this would be enhanced by combining the symptom reports of a patient to other eHealth apps sensing for example metabolic or physiologic changes, is another fascinating possibility [38].

## Conclusions

The current study suggests that ML based prediction models using ePRO and EHR data as an input can predict the presence and onset of irAEs with a high accuracy. Thus, it indicates that digital symptom monitoring combined with ML could facilitate the detection of irAEs in ICI-treated cancer patients.

## Data Availability

The datasets generated and/or analyzed during the current study are not publicly available but are available from the corresponding author on reasonable request.

## Abbreviations

ePRO: Electronic patient reported outcome
HCP: Health care personnel
ICIs: Immune-checkpoint inhibitors
irAEs: Immune-related adverse events
QoL: Quality of life
ML: Machine learning
CTCAE: Common terminology criteria for adverse events
AUC: Area under curve
MCC: Mathew: s correlation coefficient
EHR: Electronic health care record
AI: Artificial intelligence
XGBoost: Extreme gradient boosting algorithm
